# Resorc[4]arene-Modified Gold-Decorated Magnetic Nanoparticles for Immunosensor Development

**DOI:** 10.1021/acs.bioconjchem.2c00605

**Published:** 2023-02-08

**Authors:** Andrea Calcaterra, Francesca Polli, Lara Lamelza, Cristina Del Plato, Silvia Cammarone, Francesca Ghirga, Bruno Botta, Franco Mazzei, Deborah Quaglio

**Affiliations:** Department of Chemistry and Technology of Drugs, Department of Excellence 2018−2022, Sapienza—University of Rome, P.le Aldo Moro 5, 00185 Rome, Italy

## Abstract

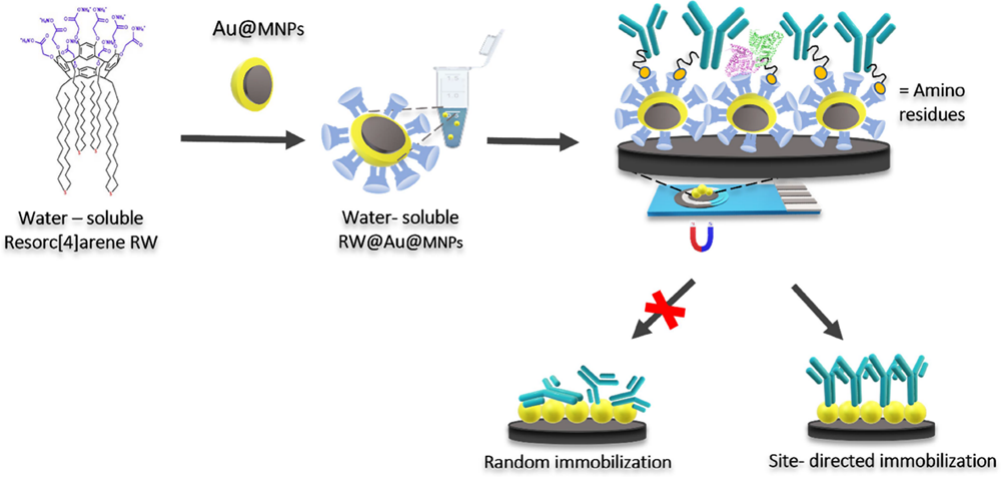

In recent years, several efforts have been made to develop selective, sensitive, fast response, and miniaturized immunosensors with improved performance for the monitoring and screening of analytes in several matrices, significantly expanding the use of this technology in a broad range of applications. However, one of the main technical challenges in developing immunosensors is overcoming the complexity of binding antibodies (Abs) to the sensor surface. Most immobilizing approaches lead to a random orientation of Abs, resulting in lower binding site density and immunoaffinity. In this context, supramolecular chemistry has emerged as a suitable surface modification tool to achieve the preorganization of artificial receptors and to improve the functional properties of self-assembled monolayers. Herein, a supramolecular chemistry/nanotechnology-based platform was conceived to develop sensitive label-free electrochemical immunosensors, by using a resorcarene macrocycle as an artificial linker for the oriented antibody immobilization. To this aim, a water-soluble bifunctional resorc[4]arene architecture (RW) was rationally designed and synthesized to anchor gold-coated magnetic nanoparticles (Au@MNPs) and to maximize the amount of the active immobilized antibody (Ab) in the proper “end-on” orientation. The resulting supramolecular chemistry-modified nanoparticles, RW@Au@MNPs, were deposited onto graphite screen printed electrodes which were then employed to immobilize three different Abs. Furthermore, an immunosensor for atrazine (ATZ) analysis was realized and characterized by the differential pulse voltammetry technique to demonstrate the validity of the developed biosensing platform as a proof of concept for electrochemical immunosensors. The RW-based immunosensor improved Ab_ATZ_ loading on Au@MNPs and sensitivity toward ATZ by almost 1.5 times compared to the random platform. Particularly, the electrochemical characterization of the developed immunosensor displays a linearity range toward ATZ within 0.05–1.5 ng/mL, a limit of detection of 0.011 ng/ml, and good reproducibility and stability. The immunosensor was tested by analyzing spiked fortified water samples with a mean recovery ranging from 95.7 to 108.4%. The overall good analytical performances of this immunodevice suggest its application for the screening and monitoring of ATZ in real matrices. Therefore, the results highlighted the successful application of the resorc[4]arene-based sensor design strategy for developing sensitive electrochemical immunosensors with improved analytical performance and simplifying the Ab immobilization procedure.

## Introduction

A biosensor is a sensing device based on coupling a biotransducer (enzyme, antibody, DNA, etc.) and a physico-chemical transducer (electrochemical, optical, piezoelectrical, etc.). Among the most widely used biosensors, electrochemical immunosensors show high selectivity, sensitivity, miniaturizability, low cost, and fast measurements.^1,2^ Due to the heterogeneous mechanism of antibody–antigen interaction, the antibody (Ab) immobilization procedure is a crucial aspect of optimizing immunosensor performance in terms of ligand loading and antigen sensitivity. Most immobilizing approaches^3−5^ lead to a random orientation of Abs, resulting in lower binding site density and immunoaffinity. In this context, supramolecular chemistry has emerged as a suitable surface modification tool to allow the preorganization of artificial receptors and improve the functional properties of self-assembled monolayers (SAMs).^6−8^ Among the large pool of supramolecular macrocycles available, resorc[4]arenes, belonging to the family of calixarenes, are characterized by a unique three-dimensional surface that can be functionalized at both the upper and lower rims with several functional groups to tailor their recognition properties toward a specific class of analytes.^6,9^ Importantly, they are chemically stable, structurally preorganized, easy to functionalize, and available in high purity and substantial quantities.^10,11^ Recently, we designed and synthesized several resorc[4]arenes as supramolecular artificial linkers for oriented antibody immobilization.^12^ In this previous work, we demonstrated that a gold surface plasmon resonance sensor chip surface modification by suitably functionalized resorc[4]arene macrocycles represents a potentially powerful system to improve sensitivity, providing new insight into sensor development.^13^ However, the sensitivity of optical methods follows the well-known Lambert–Beer law, and minimum sample volume and path length are required to achieve certain performances. The electrochemical methods appear as a promising alternative to optical approaches providing good precision, accuracy, and sensitivity with relatively simple instrumentation.^13^ Miniaturized dimensions and large-scale production characterize screen-printed electrodes (SPEs). The use of graphite instead of other electrode materials is due to its low cost, conductivity, and chemical and electrochemical stability. Furthermore, graphite surfaces can be easily modified with suitable nanomaterials, particularly gold-based nanoparticles, which are absorbed on the carbon surface throughout charge interaction. Indeed, these features make the electrochemical approach more appealing for high-throughput analysis compared to traditional diagnostics.^14^ Graphitic materials (such as graphite, glassy carbon, and nanographite) offer outstanding conductivity, chemical and electrochemical stability, versatility, wide potential windows, and rich surface chemistry.^15^ Depending on different types of targets for detection, they can be modified with suitable nanomaterials to improve their stability and effectiveness.^16^ In particular, gold-coated magnetic nanoparticles (Au@MNPs) have been deemed charming due to their high surface-to-volume ratios and their enhanced analytical performance with respect to other designs. Indeed, Au@MNPs can be used for several applications due to their high versatility. The optical and magnetic properties of the particles can be tuned and tailored to applications by changing their size, gold shell thickness, shape, charge, and surface modification.^17^ The introduction of macrocyclic molecules to the Au@MNPs nanomaterial surface provided a novel path for the fabrication of versatile and diverse hybrid nanomaterials, which combined and enhanced the characteristics of the two components.^18−20^

Herein, considering the advantages of SPEs, Au@MNPs, and resorcarene-based linkers, we have integrated them in the fabrication of electrochemical immunosensors to achieve high performance.

**Scheme 1:**
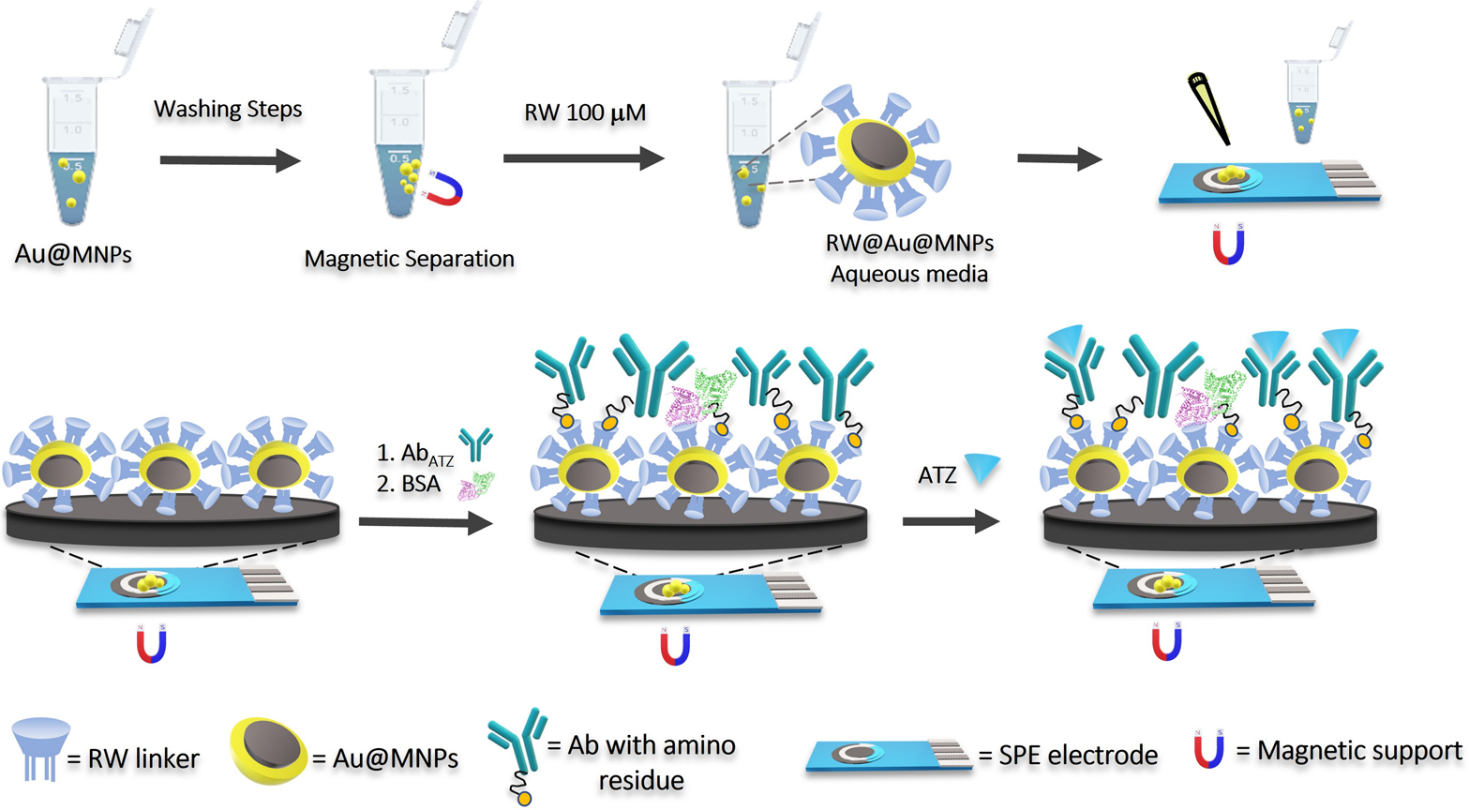
Scheme of Immunosensor Assembly with the Resorcarene Molecules Highlighted in Light Blue The figure shows all the stepwise modifications: functionalization, washing procedure, dropcasting, antibody immobilization, and antigen interaction.

To this aim, a bifunctional resorc[4]arene derivative RW, decorated at the upper rim with eight hydrophilic carboxylate groups and featuring long thioether alkyl chains at the lower rim, was designed and synthesized. Accordingly, a graphite SPE was modified with Au@MNPs functionalized with the resorc[4]arene derivative allowing the site-direct orientation of Abs, hence, increasing the immunosensor sensitivity. A schematic representation of the immunosensor assembly is shown in Scheme 1. The Au@MNPs functionalization was ensured by the binding between the thioether groups on the lower rim of the resorc[4]arene derivative and the gold surface of Au@MNPs. The upper rim properly functionalized is involved in the Ab constant fragment (Fc) interaction. Indeed, the resorc[4]arene RW is soluble in water media, avoiding problems connected with using organic solvents as possible electrode surface damage and enhancing biocompatibility.

In particular, the RW@Au@MNPs system was employed to immobilize three different monoclonal antibodies: anti-progesterone antibody (Ab_Pg_), Spike protein S1 antibody (Ab_SPS1_), and anti-Atrazine antibody (Ab_ATZ_). Ab density and immunosensor sensitivity improvement was assessed by comparing the RW-based approach with those obtained by Ab random immobilization using EDC/NHS cross-linking. These experiments have been realized to demonstrate that the RW-based immobilization can be used for different proteins with a good immobilization yield. This aspect is particularly useful in immunosensor fabrication where the most commonly used immobilization techniques have to be optimized on each single antibody feature (i.e., isoelectric point).

Furthermore, to confirm the technological platform’s validity, an Ab_ATZ_-based electrochemical immunosensor was developed and characterized in ATZ standard solutions and water samples fortified with ATZ.^21^ This study outlines the successful application of the resorc[4]arene-based sensor design strategy to develop label-free and miniaturized electrochemical immunosensors. Indeed, the resorc[4]arene-based immunosensor resulted in a versatile platform that can be adequately modified, extending its application to different biotransducers.

## Results and Discussion

### Design and Synthesis of the Resorc[4]arene Macrocycle RW To Improve Au@MNPs-SPEs Selectivity toward Abs

In recent years, Au@MNPs nanomaterials have attracted great interest in the construction of biosensing devices due to their outstanding properties. The ability to modify the nanoparticles surface in a controllable manner on a molecular level is important to impart specificity, sensitivity and biological compatibility to Au@MNPs.^17^ Macrocyclic compounds (i.e., cyclodextrins, cucurbit[*n*]urils, calixarenes, and pillar[*n*]arenes) could be used as stabilizing capping agents for the preparation of nanomaterials which also enhanced their recognition and sensing capacity on the material surface.^22^ The supramolecular self-assembly of macrocycle-modified nanomaterials allows the formation of morphologically controlled or highly ordered arrays, which was an important feature for miniaturized systems. In this context, the use of modified Au@MNPs could play a key role in enhanced immunosensor development.^23−25^ Our previous work demonstrated that surface modification by properly functionalized resorcarene macrocycles allows the optimal Ab orientation favoring the “end-on” configuration.^7,12^ In this previous work, we demonstrated that the introduction at the upper rim of thioether alkyl chains allows an optimal functionalization procedure of the gold sensor disk surface by forming SAMs via oxidative absorption. However, due to the poor water-solubility of these macrocycles, most of the resorcarene based-sensors are prepared or applied in organic solvents such as tetrahydrofuran, toluene, chloroform, dichloromethane, etc., which may bring environmental pollution and severely limits their potential applications in the future.

Therefore, it is important to synthesize water-soluble calixarene and suitable electric support materials to solve these problems and then integrate calixarene supported-nanoparticles.

**Scheme 2:**
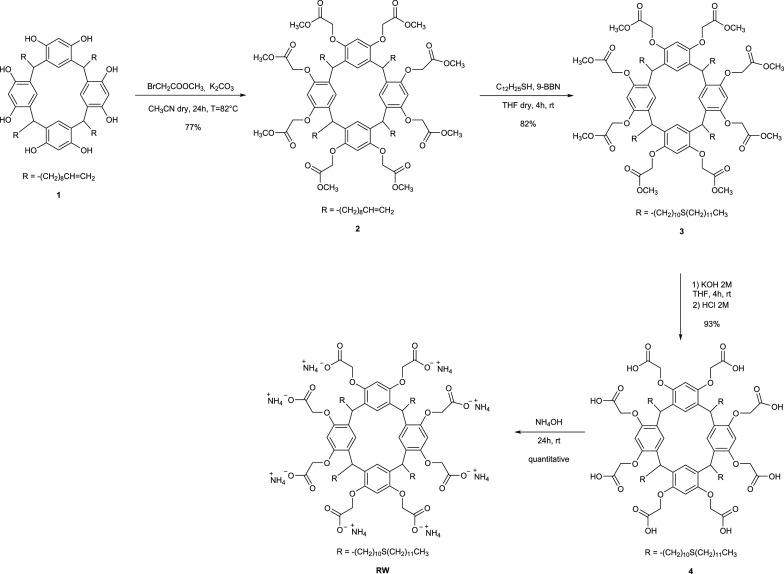
Synthesis of Resorc[4]arene-Based Linker RW

In this context, to construct a sensitive Au@MNPs-SPE-based electrochemical immunosensor, we rationally designed and synthesized a bifunctional resorc[4]arene architecture RW (Scheme 2) featuring the following structural features. The upper rim is decorated with eight hydrophilic carboxylate groups to tailor their recognition properties toward the Fc portion of Abs. Indeed, the introduction of acidic groups promotes the solubility in biocompatible and nontoxic aqueous solvents avoiding SPE degradation. Long thioether alkyl chains feature the lower rim to install the artificial linkers on the Au@MNPs covalently. The synthetic procedure to afford resorc[4]arene RW involved the thiol–ene transformation of terminal vinylidene groups and the introduction of carboxylic acid groups at the upper rim (Scheme 2). Resorc[4]arene **1** was prepared according to the literature.^26^ The phenol groups of resorc[4]arene **1** were functionalized with methyl bromoacetate in the presence of potassium carbonate as base to obtain resorc[4]arene **2**, which bears methyl ester moieties at the upper rim, in 77% yield.^27^ Successively, the resorc[4]arene octa-methyl ester **2** reacted with 1-dodecanethiol via anti-Markovnikov addition in the presence of 9-borabicyclo[3.3.1]nonane (9-BBN) as a catalyst, to obtain resorc[4]arene **3** in 82% yield.^5^ The ester functionalities of **3** were hydrolyzed with 2 M potassium hydroxide and then the solution was acidified with hydrochloric acid to obtain the resorc[4]arene octa-carboxylic acid **4** in 93% yield.^27^ Finally, upon treatment with an excess of ammonium hydroxide, the final water-soluble resorc[4]arene octa-carboxylate ammonium salt was quantitatively obtained. All macrocycles have been fully characterized by NMR and HRMS.

### RW Capping Agent Influence on Differential Pulse Voltammetry (DPV)

Au@MNPs were washed by magnetic separation and then functionalized by ligand-exchange incubating using different RW concentrations (4 mM, 2 mM, 100 μM, and 1.8 μM). Furthermore, to optimize the deposition process, each batch was drop-casted on a graphite SPE electrode at three different dilutions in water expressed in a v/v percentage between 100 and 0.10%.

The system was characterized by monitoring the current signal by DPV measurements to observe the signal amplification due to the nanomaterial as well as the effect of the capping agent used.^24,28^ As expected, the current peak decreases with increasing [RW] concentrations (Figure S7A). This phenomenon is absent in the case of unconjugated Au@MNPs, it may be addressed to the presence of RW undecanoic hydrophobic chains that obstacle the [Fe(CN)_6_]^3–/4–^ diffusion to the electrode surface, thus hindering the DPV signal amplification led by the gold layer surrounding MNPs.^29^ Indeed, higher concentrations of RW reduce the Au@MNPs stability leading to a brown background body formation on the tube walls. The presence of the RW capping agent at a concentration between 100 and 1.8 μM resulted in the best-performing functionalization procedures.

Therefore, the best concentration of the capping agent for Au@MNPs functionalization was further tested to evaluate NP stability (Figure S7B,C). In particular, the long-term stability of the RW@Au@MNPs obtained with 1.8 and 100 μM RW was investigated within 21 days by monitoring their DPV signals on the graphite SPE electrode.^30^ In Figure S7B, the lower availability of the stabilizing ligand in the 1.8 μM RW dispersion is characterized by the lowest stability time with a signal increasing up to the day 7th.^31^ This current increase could be ascribed to the formation of aggregates reducing the amount of nanoparticles available on the surface (Figure S7A, light blue columns). During the following days, as the precipitation process continues, involving more nanomaterial, the nanoparticle availability for signal amplification is so small that the signal amplification induced is reduced. On the other hand, the highest concentration of RW ligand (100 μM) stabilizing the dispersion provides a stable signal for the first 10 days, with a slight current increase occurring on day 12th. Moreover, Ab binding properties have been investigated during the following days (Figure S7C). As a result, the RW@Au@MNPs batch obtained with 100 μM RW was further studied to optimize the deposition process. To this aim, the RW@Au@MNPs were drop-casted on an SPE electrode at several dilutions between 100% (for an undiluted batch) and 1% expressed as v/v% in water (Figure S8A). The electrodes were tested by DPV in order to evaluate the samples exhibiting better signal reproducibility. This is a fundamental parameter for electrochemical immunosensor development as it heavily influences the current revealed during the immunosensor development steps. As shown in Figure S8B the best reproducibility (RSD 0.4%) has been exhibited by the undiluted batch (100% v/v) while the higher stable signal was given by the 2% dropcasting. Therefore, these batches were selected to further investigate the ability to immobilize the antibody. Indeed, the signal decrease due to protein absorption was compared. As shown in Figure S8B, a higher amount of RW@Au@MNPs on the electrode allows to increase the Ab immobilization. In fact, the higher signal decay recorded by DPV for undiluted dropcasting batches was improved thanks to a higher presence of ligands on the surface.^32^

### Ab Loading on SPE/RW@Au@MNPs

**Figure 1:**
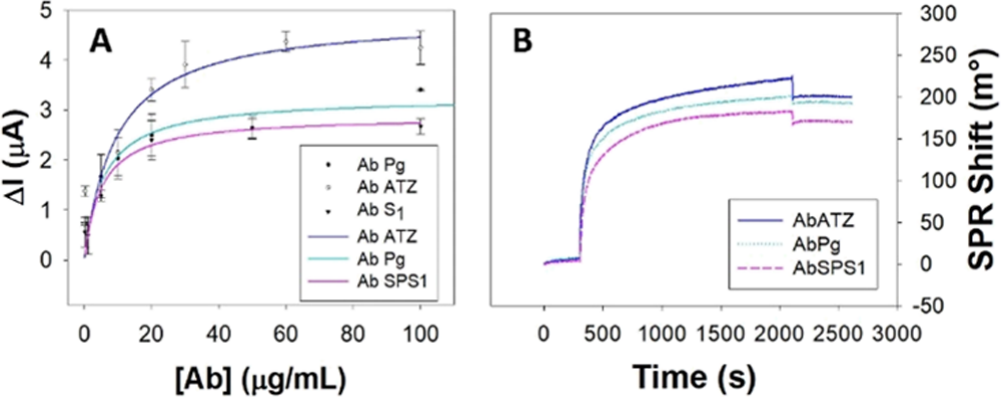
(A) DPV and (B) SPR signal obtained after Ab_SPS1_, Ab_ATZ_, and Ab_PGN_ absorption through RW immobilization.

To evaluate the loading of immobilized antibodies on the RW@Au@MNPs-modified SPEs, increasing concentrations (in the range of 0.2–100 μg/mL) of three different antibodies Ab_SPS1_, Ab_ATZ_, and Ab_Pg_ were added onto the modified electrode surface and tested by DPV. From the superimposition of the Ab adsorption isotherms in Figure 1A, it can be observed that all three antibodies saturate the electrode surface at similar concentrations (∼30–60 μg/mL). However, the current intensity decreases according to the antibody analyzed. In particular, while Ab_SPS1_ and Ab_Pg_ showed similar behavior, in the case of Ab_ATZ_ a more significant signal decay was observed.^14^ The RW-Ab interaction was also followed by the surface plasmon resonance (SPR) technique to obtain the amount of Ab immobilized on a RW functionalized gold chip in saturation conditions. However, from SPR shifts, a quite similar protein density for each Ab (Figure 1B) can be calculated according to the SPR manufacturer technical manual conversion assessing that an angle shift of 122 m° corresponds to 1 ng/mm^2^ of interacting protein. The results obtained are ranging from 140 to 160 ng/mm^2^ with a no significant SPR angle increase between Ab_Pg_ Ab_ATZ_ (Supporting Information, Table S2).^33,34^ The different behavior observed in the loading curve recorded with DPV measurements (Figure 1A) can be ascribed to the different amounts of positively charged residues of Ab proteins, partially hindering the signal decay occurring during the Ab immobilization.^35−38^ Furthermore, RW-Ab host-guest interaction involving only the Fc of the Abs structure can reduce its dependence from Abs structure characteristics^34,39^ unlike most common random immobilization procedures.

### ATZ Calibration and Real Sample Analysis

**Figure 2:**
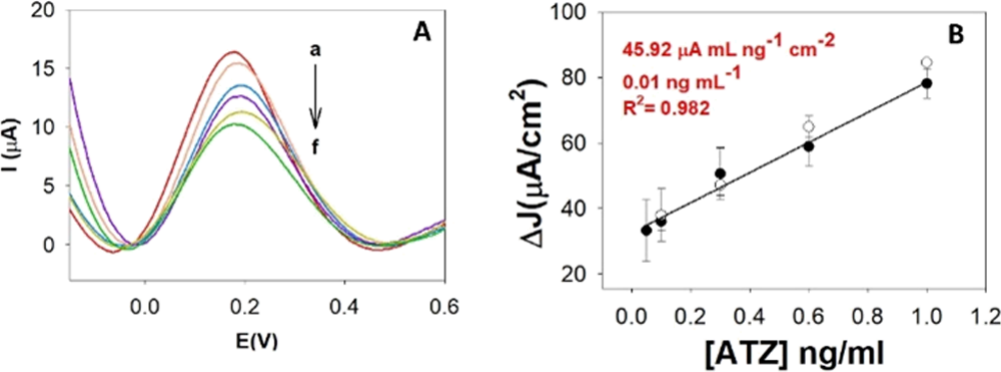
(A) Calibration plot and (B) DPV curves obtained with increasing ATZ standard solutions (2A, in black) and spiked sample values (2B, in white).

Different concentrations of ATZ, ranging from 0.05 to 10 ng/mL in PBS buffer, were tested. The current intensity was recorded during the DPV measurements (Figure 2A) assessing the anodic peak decrease occurring after ATZ interaction.^40^ The signals were plotted vs the concentration, and the calibration curve obtained is shown in Figure 2B. A linear range between 0.05 and 1.0 ng/mL and a limit of detection (LOD) of 0.01 ng/mL were obtained, making this platform suitable for ATZ detection. Furthermore, fortified water samples were analyzed in order to evaluate the matrix effect getting good results according to the recovery values ranging from 106 to 118%.

### Random vs Oriented Antibody Immobilization

To confirm the resorc[4]arene-based site-directed Ab immobilization procedure, the Ab density^36,41^ and the amount of antigen bound were compared with a random immobilization methodology using MPA@Au@MNPs-modified SPEs activated with EDC-NHS.^42^ MPA@Au@MNPs were obtained by ligand exchange^43^ and then drop-casted on a SPE electrode. The Ab random immobilization was obtained by activating the carboxylic groups of MPA with EDC/NHS coupling.^44^

**Figure 3:**
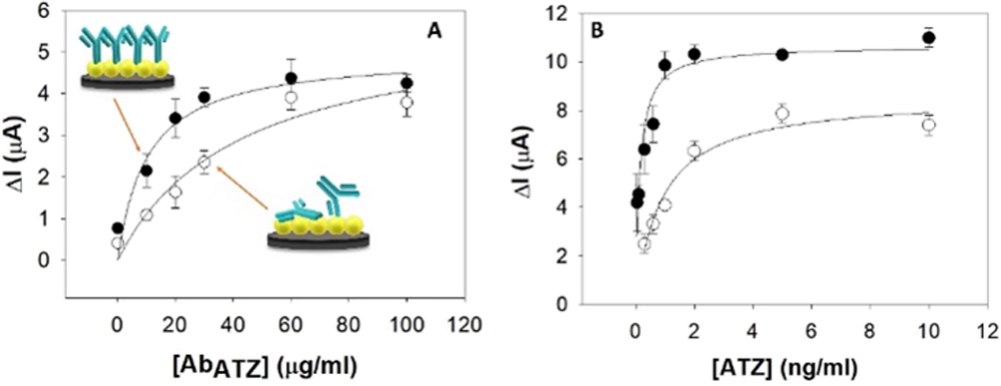
(A) Comparison of adsorption isotherms of Ab_ATZ_ in the case of oriented (in black) and random immobilization (in white). (B) Overlap of MPA and RW-based sensors (black) signals in ATZ interaction exhibiting different sensitivity and saturation signals.

Based on the Ab loading curves (Figure 3A), the Ab saturation is quite similar for both platforms, SPEs/RW@Au@MNPs and SPEs/MPA@Au@MNPs, but the resorc[4]arene-based immobilization allows to bind a more significant amount of protein at low Ab concentrations. This fact can be explained because the random immobilization procedure is generally affected by the steric hindrance caused by neighboring antibody molecules, lowering the Ab density.^23,45^ To further investigate the role of the site-direct approach of the RW platform, the calibration curve of the antigen bound by the RW@Au@MNPs-modified electrodes was compared with those obtained with the random asset SPEs/MPA@Au@MNPs. As reported in Figure 3B, the site-direct approach has shown a significantly higher sensitivity than the random configuration (∼ 46 vs ∼18 μA mL ng^–1^ cm^–2^).^42,44^ This improvement may be addressed to the optimized Ab orientation for the synergistic mechanism between host-guest interaction and dipolar momentum alignment;^7,12,34^ hence a higher population of Abs is available for the antigen-binding.^7,34^

## Conclusions

In this work, we have developed a supramolecular chemistry/nanotechnology-based platform for sensitive label-free electrochemical immunosensors. In particular, a simplification of the Ab immobilization procedure has been achieved by resorc[4]arene-modified Au@MNPs. This has been realized by the rational design and synthesis of a water-soluble bifunctional resorc[4]arene architecture RW. The bowl-shaped macrocycle RW was functionalized at the lower rim with long thioether alkyl chains to covalently install the artificial linkers on the Au@MNPs. Indeed, it was decorated at the upper rim with eight hydrophilic carboxylate groups, to tailor its recognition properties toward the Fc portion of Abs and to promote the solubility in biocompatible and nontoxic aqueous solvent avoiding the SPE degradation. The resulting supramolecular chemistry-modified nanoparticles were deposited onto graphite SPEs. The selection of the optimal concentration of RW for Au@MNPs functionalization and the stability of the RW-modified Au@MNPs were evaluated by DPV.

The immobilization process on RW@Au@MNPs-modified electrodes was evaluated for different antibodies (AbPg, AbSPS1, and AbATZ), monitoring the current signal variation by DPV. Therefore, an immunosensor for ATZ analysis was then developed and characterized to demonstrate the biosensing platform’s validity as a proof of concept for electrochemical immunosensors. This system showed a linear range of 0.05–1.5 ng/mL with an LOD of 0.01 ng/mL, which is nine times lower than the European allowed limit (0.1 ng/mL). The recoveries obtained using spiked fortified water samples provided a mean value ranging from 106 to 118%, with good reproducibility, thus suggesting the feasibility of the RW@Au@MNPs-modified SPEs for developing sensitive immunosensors for real sample analysis.

Indeed, to confirm the ability of RW to drive the site-directed immobilization of antibodies, the Ab density and the amount of antigen bound were compared with a random immobilization methodology using MPA@Au@MNPs-modified SPEs activated with EDC-NHS. Comparing the Ab loading curves, the RW-based immobilization showed an improvement of the amount of protein immobilized. In addition, the comparison of the site-directed and random-based immunosensor ATZ/Ab_ATZ_ association curves evidenced the better analytical performance of the RW-based immunosensor in terms of sensitivity improvement, lower LOD, and extended linearity range.

Therefore, these results demonstrated that the supramolecular antibody conjugation strategy based on resorc[4]arene modifiers resulted in a versatile platform that can be adequately modified, extending its application to different biotransducers to develop label-free and miniaturized electrochemical immunosensors.

## Experimental Section

### Materials

All solvents and reagents were purchased from Merck KGaA (Darmstadt, Germany) or Carlo Erba Reagents, (Milano, Italy) and used without further purification unless otherwise stated. Melting points were recorded with a certified Büchi melting point B-545 apparatus in open capillaries. NMR spectra have been acquired with a Bruker Avance/Ultra ShieldTM 400 spectrometer operating at 400.13 MHz for ^1^H and 100.62 MHz for ^13^C at room temperature, using 5 mm diameter glass tubes. Chemical shifts (δ) are reported in parts per million (ppm) and coupling constants (*J*) in hertz (Hz), approximated to 0.1 Hz. The residual solvent peak was used as an internal reference for ^1^H and ^13^C NMR spectra and is referenced to CDCl_3_ (δ = 7.26 ppm for ^1^H, δ = 77.16 ppm for ^13^C). Data for ^1^H NMR are reported as follows: chemical shift, multiplicity (br = broad, ovrlp = over-lapped, s = singlet, d = doublet, t = triplet, q = quartet, m = multiple, dd = doublet of doublets), coupling constant, integral. All ^13^C NMR spectra were obtained with complete proton decoupling. Spectra were processed with the program MestReNova version 12.0.0–20,080, FT and zero filling at 64 K. Chromatography was carried out on 60 Å silica gel (40–63 μm, 230–400 mesh). All reactions were monitored by thin-layer chromatography (TLC), and 60 Å silica gel on TLC plates was used. High-resolution mass spectra (HR-MS) were acquired on a Bruker BioApex Fourier transform ion cyclotron resonance mass spectrometer.

Potassium ferricyanide K_3_[Fe(CN)_6_], potassium ferrocyanide K_4_[Fe(CN)_6_], potassium chloride (KCl); sodium hydroxide (NaOH); 0.5 M hydrochloric acid (HCl), dibasic sodium hydrogen phosphate (Na_2_HPO_4_), monobasic sodium hydrogen phosphate (NaH_2_PO_4_), 2-(*N*-morpholin)ethanesulfonic acid(MES), 1-ethyl-3-(3-dimethyl-aminopropyl) carbodiimide (EDC), *N*-hydroxysuccinimide (NHS), ethanolamine (NH_2_CH_2_CH_2_OH), bovine serum albumin (BSA), 3-mercaptopropionic acid (MPA), and monoclonal anti-progesterone antibody (Ab_Pg_) were purchased from Sigma-Aldrich (St. Louis, MO, USA). Anti-Atrazine antibody (Ab_ATZ_) was purchased from Agrisera (Sweden) while Spike protein S1 antibody (Ab_SPS1_) and Spike S1 protein were purchased from Sinobiological (USA). Screen-printed graphite electrodes (DRP-110) and their magnetic support were purchased from Metrohm (Switzerland) while the magnetic rack was obtained by Adem-Tech (France). Gold-recovered magnetic nanoparticles (Au@MNPs) were obtained by Micromod (GmbH). According to the manufacturer features, they exhibit a size of 250 nm, a polydispersity index <0.2, a pH ranging from 7.0 to 9.0 at 25 mg/mL (20 °C), with 5.7 × 10^11^ nanoparticles per ml, and a density of 5.35 g/ccm. The batch has a magnetization of 46 Am^2^/kg iron (H = 80 kA/m) and a saturation magnetization: >71 Am^2^/kg iron (H > 800 kA/m) with a coercive field Hc: 0.481 kA/m.

#### Resorcarene Preparation and Characterization

Resorc[4]arene **1** was synthesized according to the procedure previously reported, and the chemical structure was confirmed based on reported data.^26,46^

Resorc[4]arene **2**

To a solution of **1** (0.96 mmol, 1 g) in acetonitrile (CH_3_CN) (50 mL) were added methyl bromoacetate (BrCH_2_COOCH_3_) (9.6 mmol, 1.47 g) and potassium carbonate (K_2_CO_3_) (19.2 mmol, 2.65 g). The mixture was heated at 80 °C under nitrogen gas protection for 24 h. Then, the reaction mixture was cooled to room temperature and concentrated under vacuum. The crude product was resuspended with dichloromethane (CH_2_Cl_2_), washed first with a 1 N solution of acid chloride (HCl) (1 × 70 mL) and then with brine (2 × 70 mL), and dried over anhydrous Na_2_SO_4_, and the solvent was removed under reduced pressure. The crude product was resuspended with methanol (MeOH), and the solution was stirred at room temperature for 12 h. The precipitate was filtered, and the filtrate was concentrated, giving resorc[4]arene **2** as a white solid in 77% yield.

m.p. 268 ± 0.5 °C. ^1^H NMR (CDCl_3_, 400 MHz): δ (ppm) = 6.60 (s, 4H, Ar*H*_ext._), 6.20 (s, 4H, Ar*H*_int._), 5.86–5.71 (m, 4H, RC*H*=CH_2_), 4.96 (d, *J* = 17.1 Hz, 4H, RCH=C*H*_2_), 4.90 (dd, *J* = 10.2, 0.9 Hz, 4H, RCH=C*H*_2_), 4.58 (t, *J* = 7.4 Hz, 4H, ArC*H*Ar), 4.21 (s, 16H, ArOC*H*_2_CO), 3.68 (s, 24H, C*H*_3_OCOR), 2.06–1.96 (m, 8H, RC*H*_2_CH=CH_2_), 1.90–1.77 (m, 8H, RC*H*_2_CHAr_2_), 1.40–1.15 (m, 48H, C*H*_2_). ^13^C NMR (CDCl_3_,100 MHz): δ (ppm) = 169.94, 154.60, 139.35, 128.62, 126.68, 114.20, 100.87, 67.26, 52.05, 35.81, 34.63, 33.96, 30.08, 29.82, 29.80, 29.36, 29.11, 28.17.

ESI-HRMS (positive) *m*/*z*: [M + Na]^+^ C_92_H_128_O_24_Na calcd. 1639.8693; found 1639.8689.

Resorc[4]arene **3**. To a solution of resorc[4]arene **2** (0.618 mmol, 1 g) in tetrahydrofuran (THF) dry (54 mL) was added under argon 1-dodecanthiol (14.09 mmol, 2.85 g) and 9-BBN solution in 0.5 M THF (24.75 mmol, 3 g) at 0 ° C. The mixture was stirred at room temperature for 12 h. Then, the solution was concentrated under reduced pressure, the resulting solid was recrystallized from methanol (MeOH), and white crystals were obtained in 82% yield.

m.p. 290 ± 0.5 °C. ^1^H NMR (CDCl_3_, 400 MHz): δ (ppm) = 6.59 (s, 4H, Ar*H*_ext._), 6.20 (s, 4H, Ar*H*_int._), 4.57 (t, *J* = 7.4 Hz, 4H, ArC*H*Ar), 4.27 (s, 16H, ArOC*H*_2_CO), 3.75 (s, 24H, C*H*_3_OCOR), 2.53–2.39 (m, 16H, −C*H*_2_–S–C*H*_2_−), 1.92–1.75 (m, 8H, C*H*_2_), 1.67–1.46 (m, 16H, C*H*_2_), 1.39–1.19 (m, 128H, C*H*_2_), 0.87 (t, *J* = 6.7 Hz, 12H, C*H*_3_). ^13^C NMR (CDCl_3_,100 MHz): δ (ppm) = 169.94, 154.61, 128.62, 126.68, 100.89, 67.27, 52.05, 35.82, 34.63, 32.35, 32.05, 30.11, 29.96, 29.94, 29.91, 29.88, 29.83, 29.80, 29.77, 29.76, 29.69, 29.53, 29.49, 29.43, 29.21, 29.13, 28.20, 22.82, 14.26.

ESI-HRMS (positive) *m*/*z*: [M + Na]^+^ C_140_H_232_O_24_S_4_Na calcd. 2448.5714; found [M + Na]^+^ 2448.5731.

Resorc[4]are **4**

A solution of resorc[4]are **3** (0.124 mmol, 300 mg) in THF (17 mL) was treated with a 2 M aqueous solution of potassium hydroxide KOH (7.5 mL) at room temperature for 4 h. Then, the reaction mixture was evaporated under vacuum, diluted with water, and acidified with a 2 M aqueous HCl solution. The resulting precipitate was filtered, washed with water, and dried to afford resorc[4]are **4** as a white powder in 93% yield.

^1^H NMR (CDCl_3_:CD_3_OD = 98:2, 400 MHz): δ (ppm) = 7.34 (s, 2H, Ar*H*_ext._), 6.64 (s, 2H, Ar*H*_ext._), 6.16 (s, 4H, Ar*H*_int._), 4.51 (t, *J* = 7.1 Hz, 4H, ArC*H*Ar), 4.44–3.98 (m, 16H, ArOC*H*_2_CO), 2.48–2.34 (m, 16H, C*H*_2_), 1.78 (br s, 8H, C*H*_2_), 1.56–1.43 (m, 16H, C*H*_2_), 1.37–1.10 (m, 128H, C*H*_2_), 0.83 (t, *J* = 6.6 Hz, 12H, C*H*_3_). ^13^C NMR (CDCl_3_:CD_3_OD (98.2),100 MHz): δ (ppm) = 170.12, 154.45, 128.52, 126.59, 100.84, 67.14, 35.56, 34.61, 32.23, 31.95, 30.01, 29.84, 29.81, 29.77, 29.73, 29.70, 29.67, 29.65, 29.58, 29.42, 29.38, 29.31, 29.09, 29.01, 28.06, 22.72, 14.12.

ESI-HRMS (negative) *m*/*z*: [M – 2H]^2–^ C_132_H_216_O_24_S_4_ calcd. 1155.7209; found 1155.7219.

Resorc[4]arene **RW**. Resorc[4]are **4** (0.043 mmol, 100 mg) and ammonium hydroxide aqueous solution (25–28%, 30 mL) were stirred at room temperature for 24 h. Water was removed by rotary evaporation to afford resorc[4]arene **RW** as a white powder in quantitative yield.

#### RW@Au@MNPs and MPA@Au@MNP Preparation

Au@MNPs were functionalized by ligand exchange using RW and MPA solutions. The RW@Au@MNP functionalization was then optimized by varying the RW concentration. Au@MNPs were previously washed three times by magnetic separation. To this aim, 10 microL of nanoparticles were diluted with 490 μL of distilled water and left for 2 min in a magnetic rack removing the supernatant each time.^28,47^ After washing, the Au@MNPs were dispersed in 500 μL of RW solution (4 mM, 2 mM, 100 μM, and 1.8 μM), collected in a rotating agitator, and allowed to react overnight. At the end of the functionalization process, the RW@Au@MNPs were washed twice and drop-cast on the electrode at different dilutions (100% - 0.1%). The same procedure was followed for MPA functionalization by incubating the Au@MNPs in a 230 mM MPA solution in PBS buffer.^48^

#### RW@Au@MNP DPV Characterization

Au@MNPs were functionalized using several RW concentrations (4 mM, 2 mM, 100 μM, and 1.8 μM), and each batch was drop-cast on a graphite SPE electrode at three different dilutions (100a1, and 0.10%). DPV measurements were employed to characterize the modified electrodes in a [Fe(CN)_6_]^3–/4–^, 100 mM KCl solution.

#### Site-Directed Ab Immobilization on SPE/RW@Au@MNPs

The SPE graphite electrode was placed on the magnetic support and modified by drop-casting 15 μL of RW-functionalized Au@MNPs. The magnet presence allows the nanoparticles to create a homogeneous layer avoiding the “*coffee ring*” effect.^49^ Once the surface was dry, the SPE/RW@Au@MNP electrodes were further modified by deposition of 15 μL of a 20 μg/mL Ab solution for 30 min, rinsing the excess with PBS buffer. Next, a 0.1 mg/mL BSA solution was incubated on the electrode for 20 min to deactivate the RW molecules not involved in the antibody binding, thus avoiding unspecific antigen binding on the sensor surface.^50^ The surface was then conditioned with 10 mM PBS buffer pH 7.4 for the antigen interaction (Scheme 1).

##### SPR Measurements

SPR measurements were carried out with an Eco Chemie Autolab SPR system (Eco Chemie, The Netherlands) with a 670 nm laser diode and a vibrating mirror to modulate the angle of incidence on the sensor chip in the cuvette. The planar gold SPR disks were purchased from Xantec Bioanalytics (Germany). The gold sensor disks (25 mm in diameter) were mounted on the hemicylindrical lens (with index-matching oil) to form the base of the cuvette. An automatized pump pipetting system provides a constant mixing and sample dispensing during measurements. The cuvette temperature was carefully maintained at 25 ± 1 °C by using a Julabo thermostat. Data were recorded using a Windows pc and analyzed using Kinetic Evaluation software (EcoChemie).

Preparation to investigate the immobilized antibody density was carried out by the RW site-direct method. To this aim, a gold SPR disk was incubated overnight in a RW 1 mM aqueous solution. The sensor chip was then gently rinsed with water and dried under a nitrogen stream.^12^ The surface was stabilized in a 10 mM PB buffer pH 7.4, and then the surface was treated with 50 μg/mL Ab_ATZ_, Ab_SPS1_, and Ab_Pg_ solutions to study the RW-Ab interaction at saturation conditions.^51^

#### Random Ab Immobilization (SPE/MPA@Au@MNPs) Platform

The RW-based Abs immobilization was compared with that obtained with the random immobilization technique, to evaluate the RW capacity to promote site-directed immobilization. The Ab random immobilization platform was designed by modifying a SPE graphite electrode with an MPA@Au@MNPs hence performing the EDC/NHS cross-linking.^42^ 15 μL of MPA@Au@MNPs were drop-cast on the SPE graphite electrodes for this aim. Afterward, 15 μL of a freshly prepared EDC/NHS mixture were incubated on the electrode surface for 15 min to activate the MPA carboxylic terminal groups. After rinsing the reagent excess with 10 mM MES buffer pH 5.4, the electrode was incubated with a 20 μg/mL Ab solution for 30 min. The unreacted NHS-O-ester groups were neutralized by HOCH_2_CH_2_NH_2_ 1 M (pH 8) treatment for 20 min.^21^ ATZ response was then evaluated in the range between 0.1 and 10 ng/mL (Figures 3B and S5).

#### ATZ Calibration

All the experiments were carried out at room temperature. 15 μL of ATZ solution in the range: 0.1 ÷ 1.00 ng/mL) were drop-cast onto the Ab_ATZ_/Au@MNPs for 30 min. The electrode was then washed with PB buffer 10 mM pH 7.4 to remove the nonbound atrazine.^52^

#### DPV Measurements and Electrochemical Apparatus

The measurements were performed in a three-electrode electrochemical cell with a solution of FeCN_6_^3–/4–^ 1.1 mM, 100 mM KCl in deionized water (*R* = 18.2 MΩ cm) in a potential range between [−0.4; +0.6] V. A graphite electrode and a calomel reference electrode (SCE) have been used as the counter electrode (CE) and reference electrode (RE), respectively. The redox couple [Fe(CN)_6_]^4–^]/[Fe(CN)_6_]^3–^] is a reversible electrochemical system widely described in the literature, whose oxidation occurs according to the scheme below.^53^
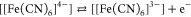


DPV curves were recorded using a μAUTOLAB potentiostat (Metrohm, The Netherlands) controlled by NOVA 2.1 software from a Windows PC.

## Supporting Information Available

^1^H and ^13^C NMR spectra, additional experiments, materials, and methods, EIS measurements, and FE-SEM characterization (PDF)
