# Supplementary material for: Resorc[4]arene-Modified Gold-Decorated Magnetic Nanoparticles for Immunosensor Development
Source: Bioconjug Chem. 2023 Feb 8;34(3):529–37. doi: 10.1021/acs.bioconjchem.2c00605 (PMC10020960; doi:10.1021/acs.bioconjchem.2c00605)
Supplement: Supplementary file 1 — bc2c00605_si_001.pdf [file bc2c00605_si_001.pdf]

Resorc[4]arene-Modified Gold-Decorated Magnetic Nanoparticles for Immunosensors Development

Andrea Calcaterra, Francesca Polli, Lara Lamelza, Cristina Del Plato, Silvia Cammarone, Francesca Ghirga*, Bruno Botta, Franco Mazzei,* Deborah Quaglio

Dr. A. Calcaterra, F. Polli, L. Lamelza, Dr. C. Del Plato, S. Cammarone, Dr. F. Ghirga, Prof. B. Botta, Prof. F. Mazzei, Dr. D. Quaglio
Department of Chemistry and Technology of Drugs
Department of Excellence 2018–2022
Sapienza – University of Rome, P.le Aldo Moro 5, 00185 Rome (Italy)
e-mail: francesca.ghirga@uniroma1.it; franco.mazzei@uniroma1.it

Table of Contents pag.

1. ^{1}H and ^{13}C NMR Spectra of Resorc[4]arenes **2**-**4** S3
2. Optimization of RW concentration for Au@MNPs functionalization S2
3. Stability of RW@Au@MNPs S3
4. Optimization of ATZ incubation time S7
5. ATZ interaction on random platform (SPE/MPA@Au@MNPs/AbATZ) S7
6. EIS measurements S8
7. FE-SEM Characterization S9
8. SPR Shifts S9

1. ^{1}H and ^{13}C NMR Spectra of Resorc[4]arenes 2-4

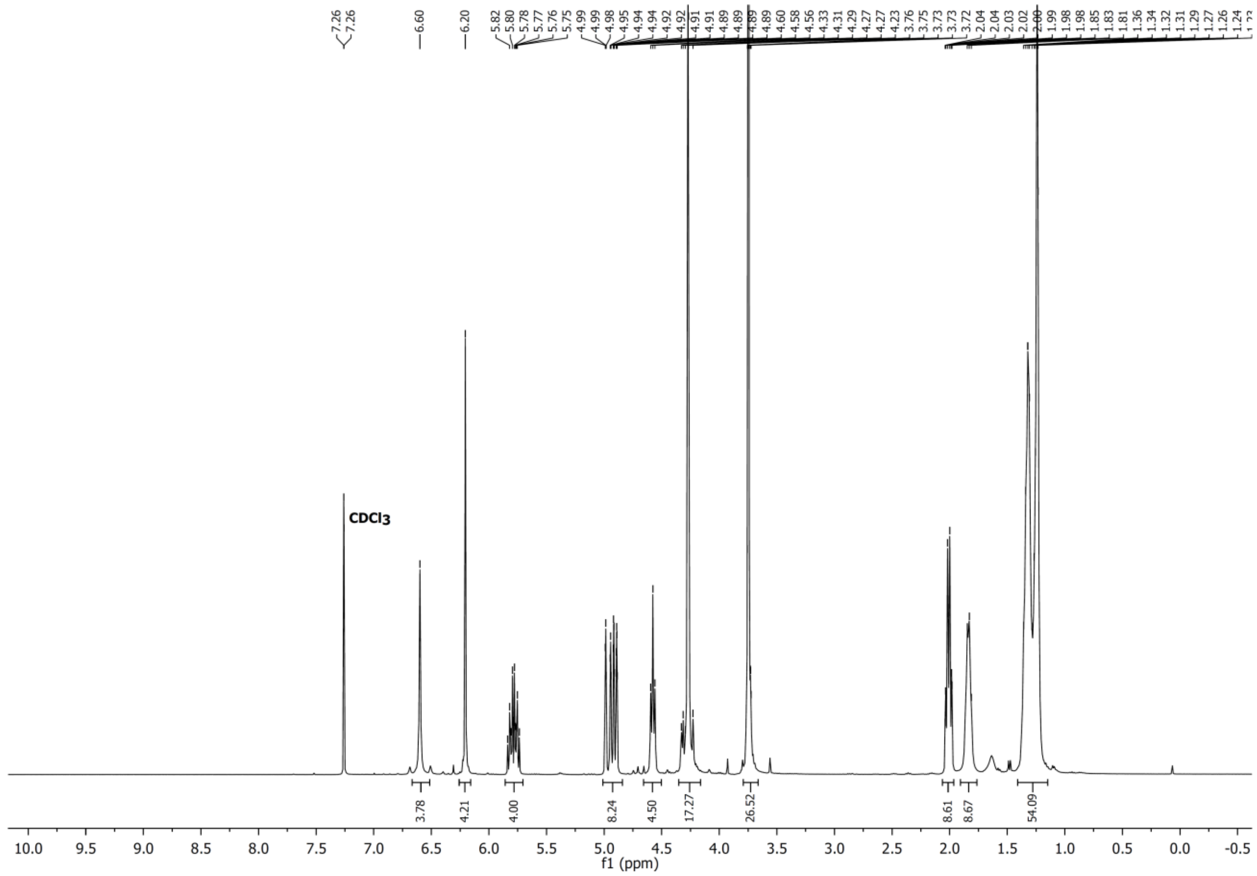

Figure S1. ^{1}H NMR Spectrum ($CDCl_3$, 400 MHz) of Resorc[4]arene **2**.

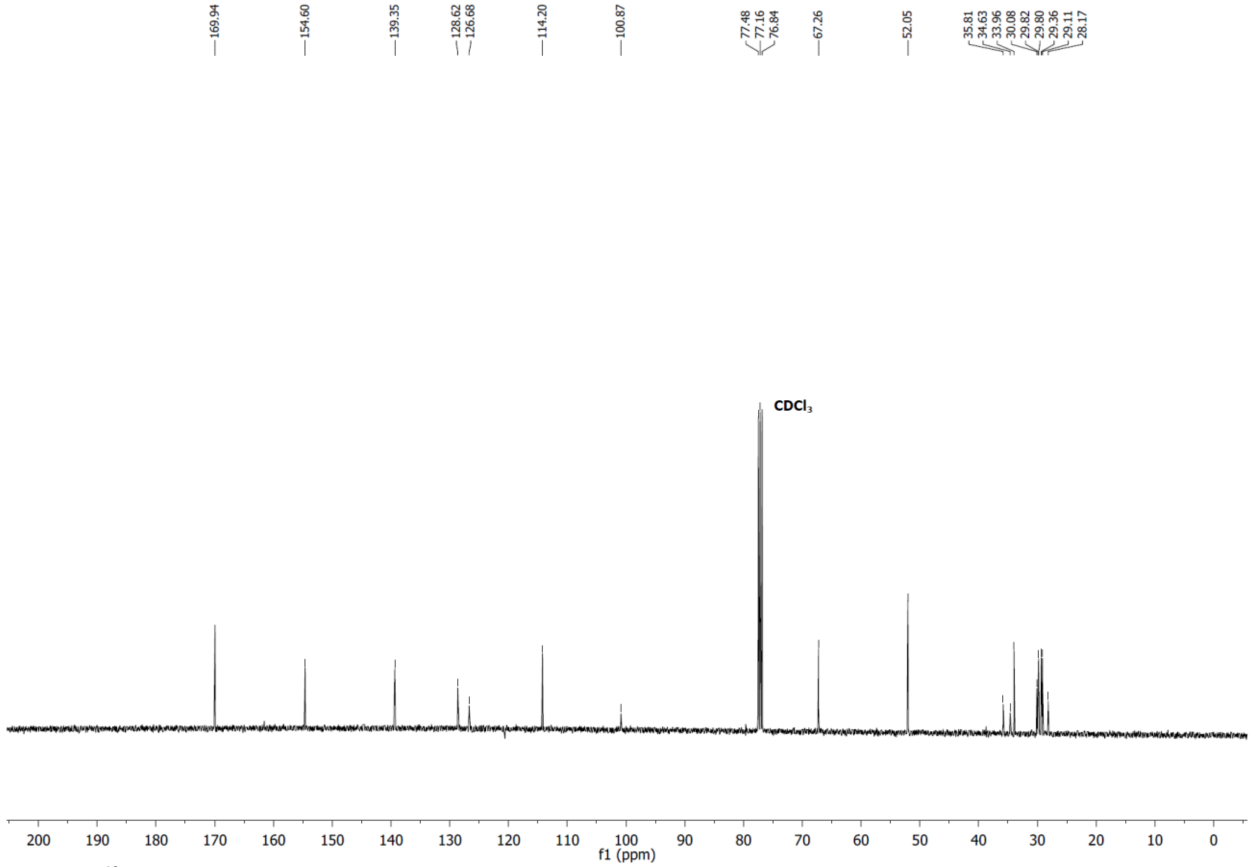

Figure S2. ^{13}C NMR Spectrum ($CDCl_3$, 100 MHz) of Resorc[4]arene **2**.

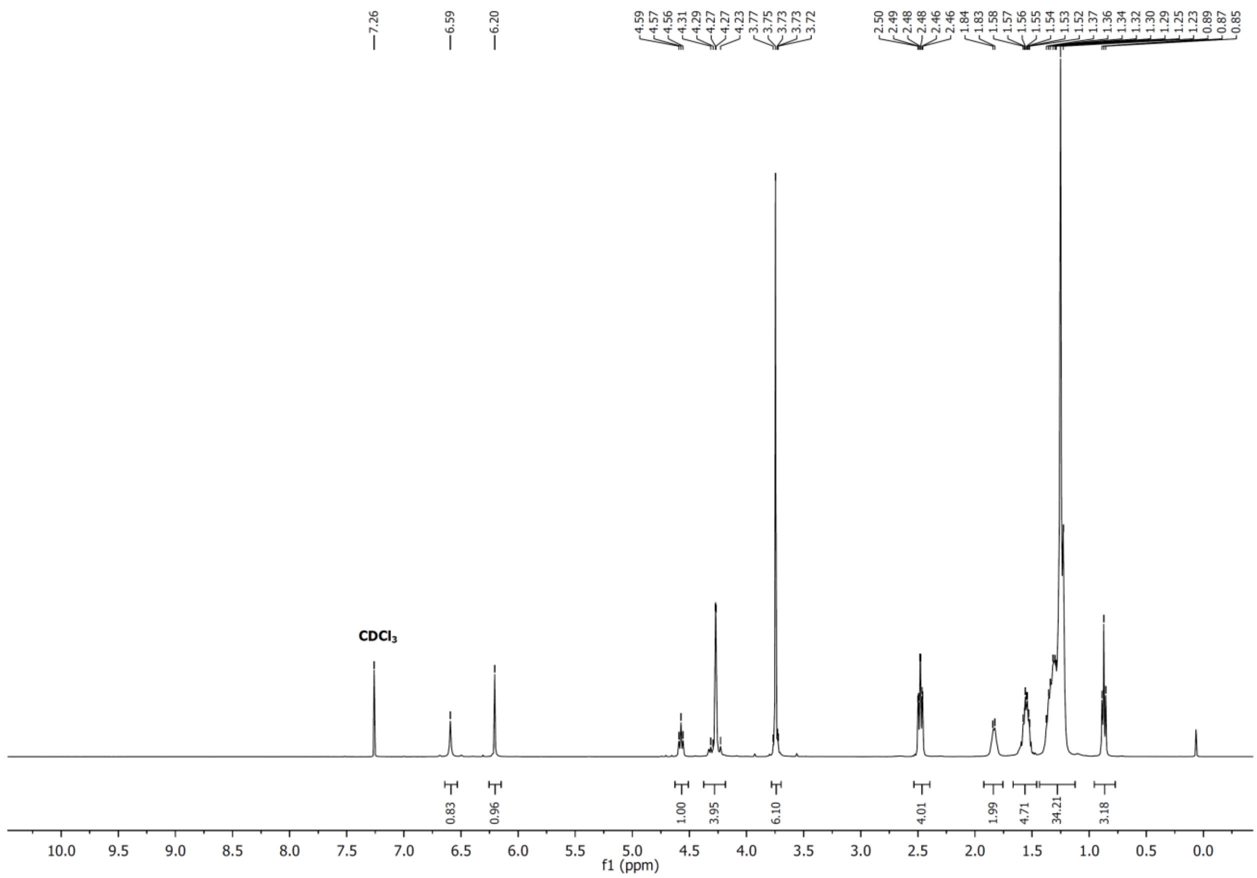

Figure S3. ^{1}H NMR Spectrum ($CDCl_3$, 400 MHz) of Resorc[4]arene **3**.

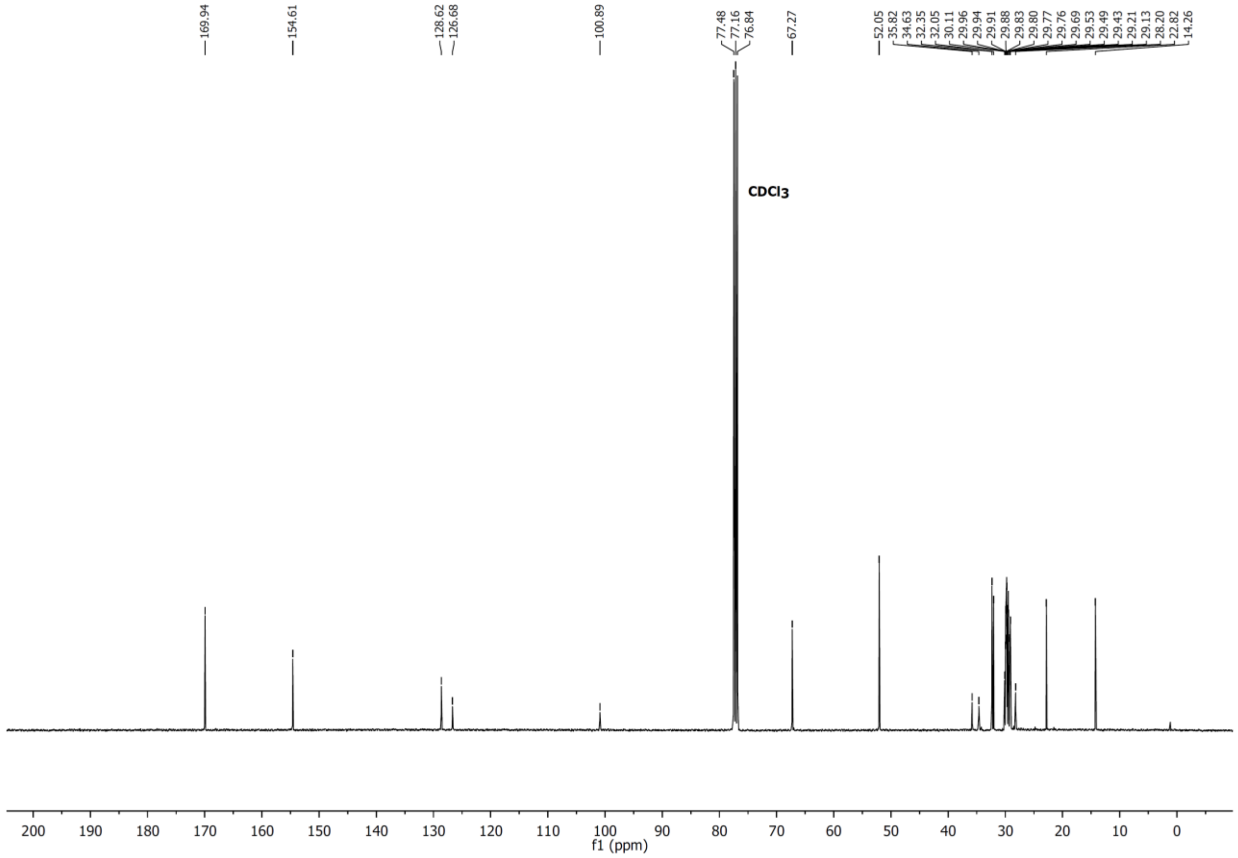

Figure S4. ^{13}C NMR Spectrum ($CDCl_3$, 100 MHz) of Resorc[4]arene **3**.

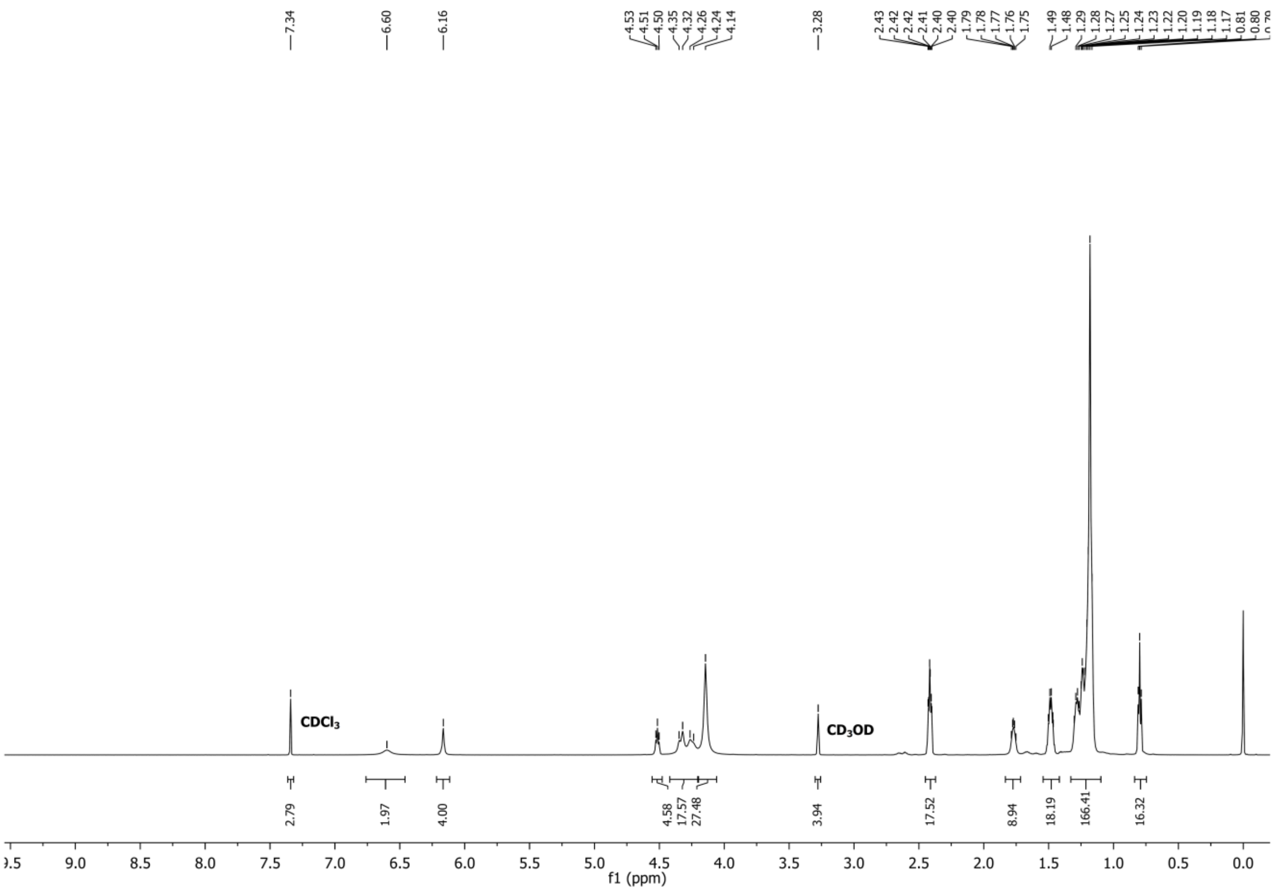

Figure S5. ^{1}H NMR Spectrum ($CDCl_3$:CD_3OD (98:2), 400 MHz) of Resorc[4]arene **4**.

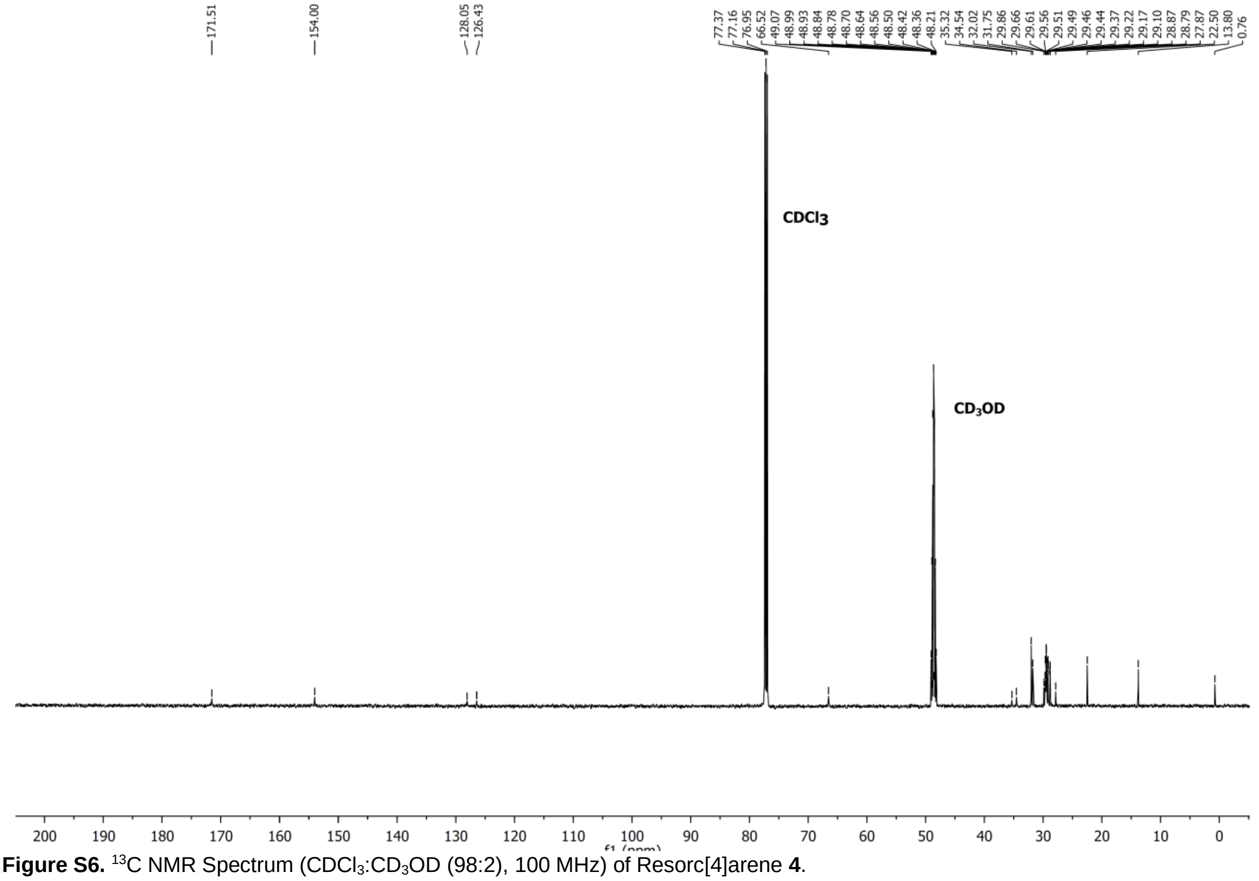

Figure S6. ^{13}C NMR Spectrum ($CDCl_3$:CD_3OD (98:2), 100 MHz) of Resorc[4]arene **4**.

2. Optimization of RW concentration for RW@Au@MNPs preparation

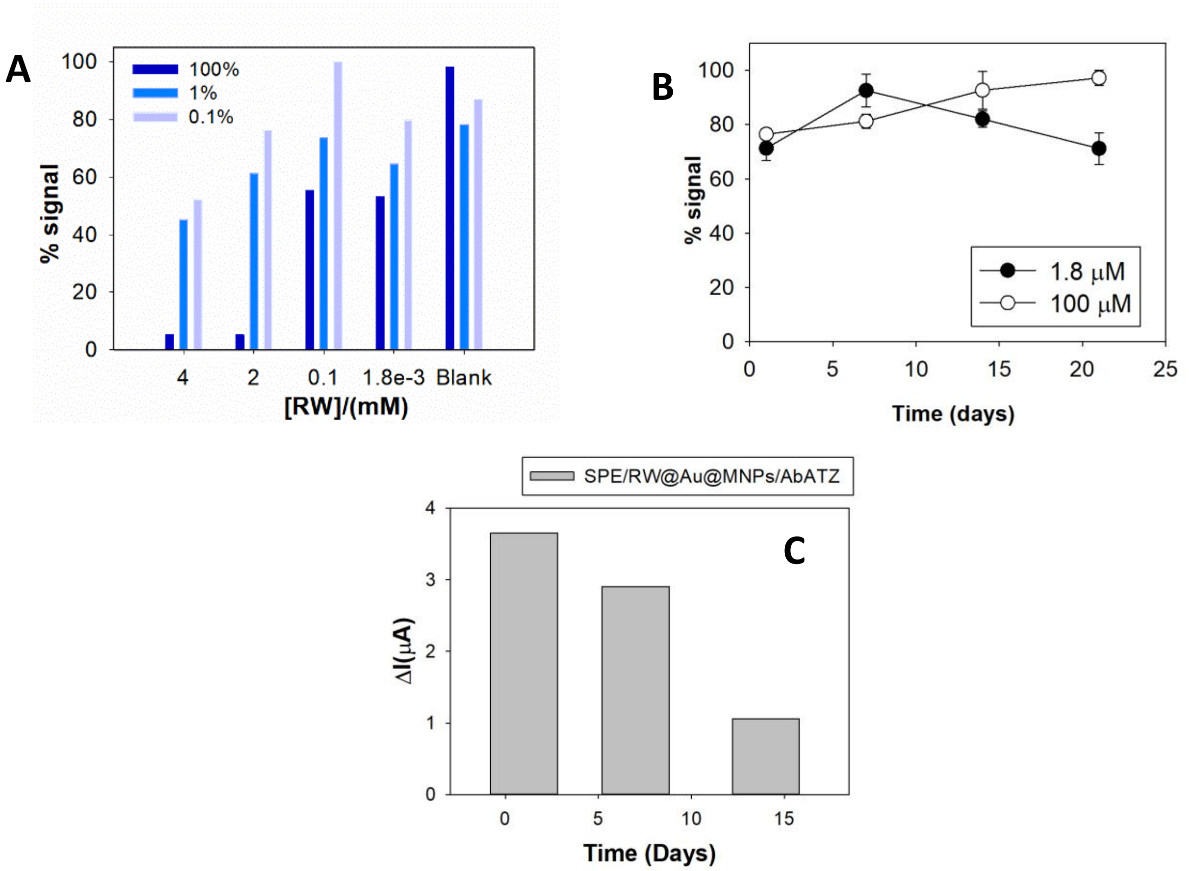

Figure S7. (**A**) DPV signals of RW@Au@MNPs prepared using different RW concentrations (4mM, 2mM, 100 µM and 1.8 µM) and dropcasted at given dilutions (100%, 1% and 0.1 %) on an SPE. (**B**) DPV signals of SPE/RW@Au@MNPs modified electrodes of batches functionalized with 1.8 µM and 100 µM of RW solution.[a] (**C**) DPV signal decrease of RW@Au@MNPs after several days from functionalization. Signal decrease obtained after Ab injection on RW@Au@MNPs after several days from functionalization.

[a] Error bars represent the standard deviation of the triplicate measurements for each time point.

3. Optimization of RW@Au@MNPs dropcasting diluition

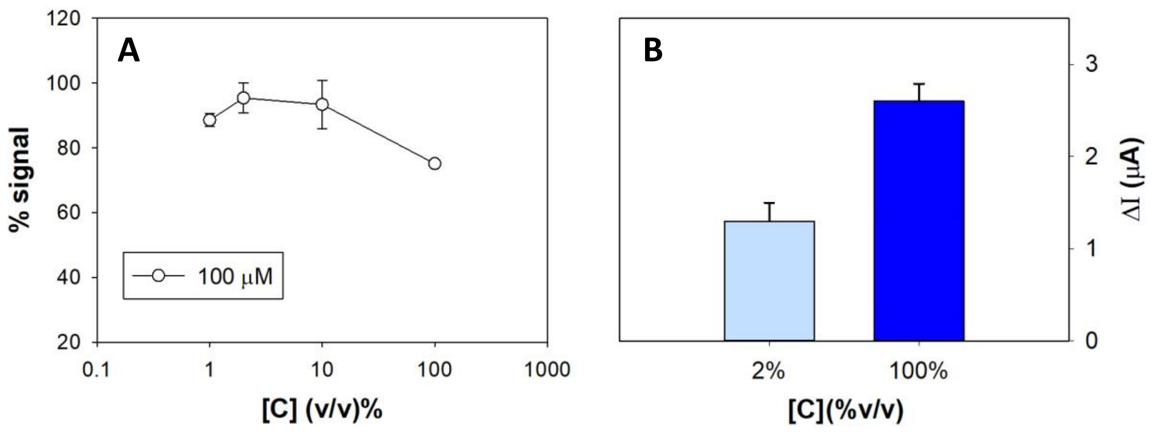

Figure S8. A) Different drop-casting dilutions (100%, 10%, 2%, 1%) on an SPE of the best-performing functionalization procedures (1.8 and 100 µM of RW). **B**) Comparison between DPV signal decrease recorded after Ab immobilization on SPE-modified electrodes modified with 2% and 100% RW@Au@MNPs.[a]

[a] Error bars represent the standard deviation of the triplicate measurements for each time point.

4. Optimization of ATZ incubation time

The antigen incubation time was optimized by monitoring the DPV signal decrease due to Ag-Ab interaction. To this aim, SPE/RW@Au@MNPs/Ab electrodes were previously deactivated with BSA and then treated with two ng/mL solution of ATZ in PBS for 15, 30, 40 and 50 minutes. The results were compared with those obtained with the negative control in PBS solution. As shown in **Figure S9** is reported the most significant signal decrease was recorded with the 30 minutes ATZ incubation.

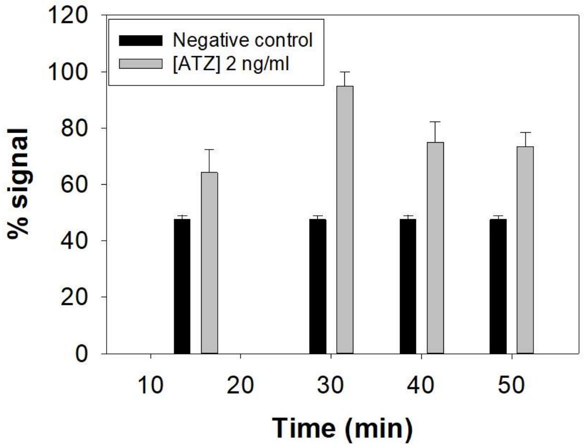

Figure S9. ATZ signals obtained with SPE/RW@Au@MNPs modified electrodes at different incubation times.[a]
[a] Error bars represent the standard deviation of the triplicate measurements for each time point.

5. ATZ interaction on random platform (SPE/MPA@Au@MNPs/Ab$_{ATZ}$)

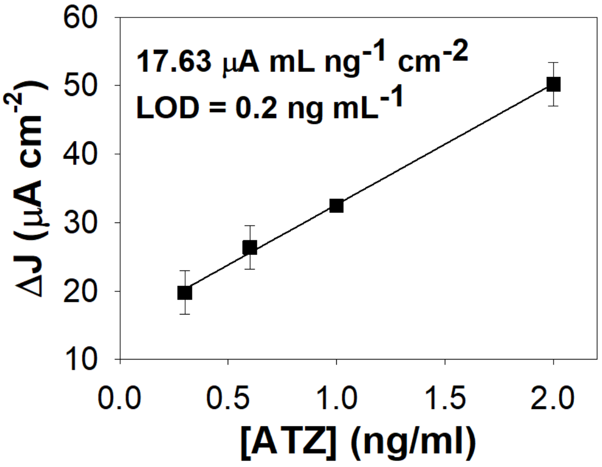

Figure S10. Calibration and sensitivity obtained with ATZ standards for the random platform (SPE/MPA@Au@MNPs/Ab$_{ATZ}$).[a]
[a] Error bars represent the standard deviation of the triplicate measurements for each time point.

6. EIS measurements

EIS measurements were taken to track stepwise modifications (**Fig. S11A**). The measurements were performed in a solution containing 5 mM [Fe(CN)6]3-/4- and 100 mM KCl was used for EIS measurements on each modified surface. To follow each modification step, EIS measurements were recorded at frequencies ranging from 10,000 to 0.1 Hz and with an amplitude of 5 mV. The measurements were carried out in a three-electrode electrochemical cell with an Ag/AgCl reference electrode and a graphite counter electrode. Moreover, the non-specific absorption on the surface was highlighted by monitoring the ATZ interaction on a BSA-modified electrode in the absence of AbATZ. To this aim, the SPE/RW@Au@MNPs electrodes were treated with 1 ng/mL of a ATZ solution (**Fig. S11B**). The Rct values were reported in Table S4. According to these findings, no significant non-specific absorption was observed.

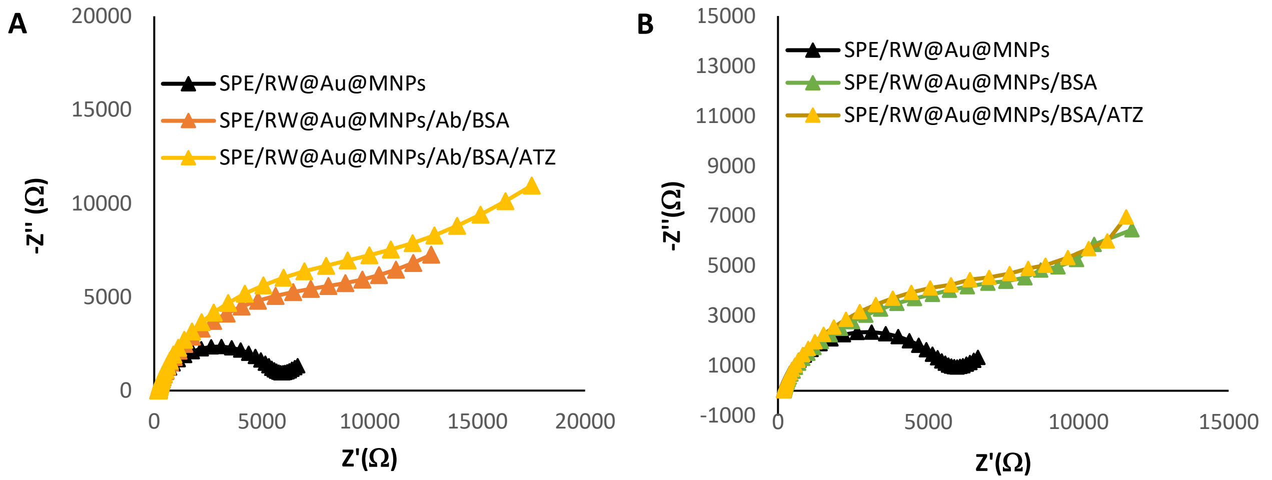

Fig.S11 EIS measurements obtained with the several SPE configurations **(A)**. Figure **(B)** reports the signal recorded after ATZ addition without AbATZ.

Tab.S1. Rct values obtained from EIS measurements of the different SPE configurations.

**Modification**	Rct **(kΩ)**
SPE	7.10
SPE/RW@Au@MNPs	5.31
SPE/RW@Au@MNPs/BSA	7.29
SPE/RW@Au@MNPs/BSA/ATZ	7.63
SPE/RW@Au@MNPs/Ab/BSA	9.52
SPE/RW@Au@MNPs/Ab/BSA/ATZ	11.2

7. FESEM characterization

High-Resolution Field Emission Scanning Electron Microscopy was used to perform scanning electron microscopy (SEM) measurements (HR FESEM, Zeiss Auriga Microscopy, Jena, Germany). The samples in **Figure S12** were prepared in accordance with the protocol outlined in the modification section. Figure B shows the SPE rim between RW@Au@MNPs tightly held by the magnet and the graphite outer rim where the nanomaterial is washed away. Figure C represents the surface after treatment with 20 µg/mL of AbATZ in PBS. Figure D, shows the surface after the saturation with BSA 0.1 mg/mL.

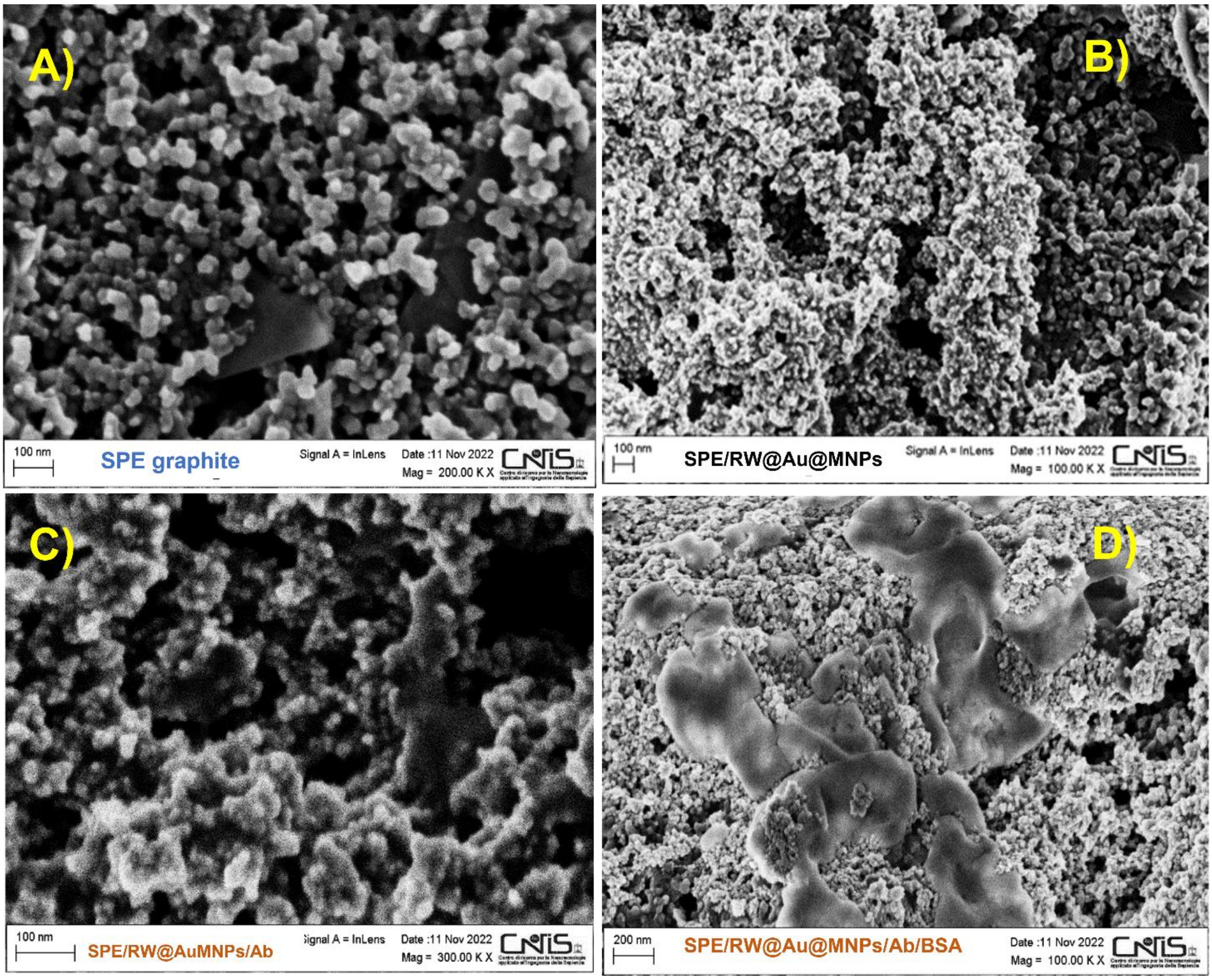

Fig.S12 SEM measurements obtained on SPE graphite electrode **(A)** modified with RW@Au@MNPs **(B)** RW@Au@MNPs@AbATZ **(C)** and RW@Au@MNPs/AbATZ/BSA **(D)**.

8. SPR Shifts

Antibody	SPR Shift *(m°)*	Protein Surface Concentration *(ng/cm²)*
**AbATZ**	193	158.2
**AbSPS1**	167	136.9
**AbPg**	187	153.3

Table S2. Surface Plasmon Resonance shifts reported in the manuscript (Figure 1B).
